# Association of lifestyle modifications with frailty in older adults: A cross-sectional study using NHANES

**DOI:** 10.1016/j.tjfa.2025.100061

**Published:** 2025-06-30

**Authors:** Yuanyuan Wu, Hongyan Peng, Rui Xu, Yingxue Hua, Yanan Zhang

**Affiliations:** a Department of Rheumatology, Luoyang Orthopedic Hospital of Henan Province (Orthopedic Hospital of Henan Province), Henan, PR China; b Xinchang Community Health Service Center, Pudong New Area, Shanghai 201314, PR China; c Faculty of Applied Sciences, Macao Polytechnic University, Macau SAR, PR China; d Outpatient Department, Taikang Tongji (Wuhan) Hospital, Wuhan 430050, PR China

**Keywords:** Frailty, Lifestyle essential 8 (LE8), NHANES, Older adults, Quantile-g-calculation

## Abstract

**Background:**

Background: Frailty significantly impacts healthy aging, yet lifestyle interventions may reduce its prevalence. This study investigated the association between a comprehensive lifestyle score, comprising eight modifiable factors (diet, physical activity, smoking, sleep, body mass index [BMI], non-HDL cholesterol, blood glucose, and blood pressure), and frailty risk in older adults, identifying key components for targeted interventions.

**Methods:**

Using data from the National Health and Nutrition Examination Survey (NHANES, 2005–2018), we analyzed 10,065 adults aged ≥60 years (mean age: 69.61, 54.97 % female). Each lifestyle factor was scored from 0 to 100, and frailty was defined using a 49-item index (>0.21). Weighted logistic regression assessed individual associations, restricted cubic spline (RCS) analysis explored dose-response relationships, and quantile g-computation evaluated joint effects.

**Results:**

Higher scores for dietary score (OR=0.69, 95 % CI: 0.52–0.90, score 100 vs. 0), non-smoking (OR=0.62, 95 % CI: 0.51–0.75), sleep (OR=0.29, 95 % CI: 0.17–0.49), blood glucose (OR=0.27, 95 % CI: 0.17–0.44), and blood pressure (OR=0.43, 95 % CI: 0.30–0.61, score 25 vs. 0) were associated with lower frailty risk (all *P* < .05). Diet and non-HDL cholesterol showed no linear association; smoking, BMI, blood glucose, and blood pressure exhibited non-linear patterns (*P*<.05). A simultaneous one-level increase in all lifestyle factors reduced frailty risk by 94 % (95 % CI: 92–95 %), with physical activity, blood glucose, and sleep as primary contributors.

**Conclusion:**

These findings highlighted the association between specific lifestyle factors and reduced frailty risk, underscoring the need for prospective studies to prioritize interventions for frailty prevention.

## 1 Introduction

With increasing life expectancy, population ageing has emerged as a pressing global challenge [1,2]. This demographic transition brings physiological decline, often leading to frailty. Frailty is characterized by impaired daily functioning, slower gait, and heightened vulnerability to stressors, affecting around 7 % of adults aged 60 and older across 62 countries [3,4]. It imposes significant burdens on individuals, families, and healthcare systems, contributing to poorer quality of life, falls, and premature mortality. Economically, frailty generates annual healthcare costs ranging from $8620 to $29,910 per older adult, with 40 % to 76 % tied to medical expenses [5]. Notably, frailty is both reversible and manageable, highlighting the importance of identifying modifiable risk factors, especially those related to lifestyle, to enable early interventions and bolster public health strategies for ageing populations [6].

The World Health Organization’s Decade of Healthy Ageing (2021–2030) underscores a global commitment to tackling these issues. This initiative seeks to create environments that promote healthy ageing, with a strong emphasis on preventing and managing age-related conditions such as frailty. Extensive research has investigated frailty’s determinants, including comorbidities, nutritional status, and lifestyle. As a modifiable factor, lifestyle shows particular promise, with evidence indicating that behaviors like regular physical activity, adequate sleep, and non-smoking can lower frailty risk. However, prior studies have limitations. Many focus on isolated lifestyle factors, overlooking their combined and interactive effects on frailty. Even when multiple factors are considered, correlations among them can obscure individual contributions, undermining the reliability of results. To overcome these shortcomings, a comprehensive lifestyle index paired with robust statistical methods is critical for a thorough assessment of lifestyle’s impact on frailty.

Previous studies have extensively explored the relationship between lifestyle behaviors and frailty, often focusing on individual factors such as physical activity, smoking cessation, or adherence to specific diets. Some research has employed composite lifestyle scores (e.g., Healthy Lifestyle Index or Mediterranean Diet Score) to capture multidimensional effects [7]. These studies generally suggest that healthier lifestyle patterns are associated with lower frailty risk. However, the analytical methods used (mostly traditional regression models) often fail to account for the interdependence and potential interactions among lifestyle components, which may lead to biased or oversimplified conclusions [8,9].

Despite the growing interest in frailty prevention, limited research has examined the association between the comprehensive Life’s Essential 8 (LE8) framework and frailty. Moreover, to our knowledge, no studies have applied quantile g-computation to assess the joint and relative contributions of LE8 components to frailty risk. This gap underscores the need for integrative approaches that can capture the complex interplay among lifestyle exposures while addressing multicollinearity and non-linear associations.

The American Heart Association’s LE8 offers a holistic framework for evaluating lifestyle, initially designed to improve cardiovascular health [10]. It includes eight components: diet, physical activity, smoking, sleep, body mass index, blood lipids, blood glucose, and blood pressure. Although its relationship with frailty is underexplored, LE8 provides a promising lens for examining lifestyle’s broader influence on this condition. To investigate this, we utilize Quantile-g-computation, an advanced statistical approach tailored to mixed exposures [11]. Unlike conventional methods, it efficiently estimates joint effects, accommodating positive, negative, or null contributions from different components without assuming linear relationships—ideal for capturing lifestyle’s complex health impacts [12].

In summary, the primary objectives of this study are: 1) To evaluate the association between individual lifestyle components of Life’s Essential 8 and frailty, including potential dose–response relationships; 2) To assess the combined effect of lifestyle factors on frailty and identify the most influential contributors using quantile g-computation. These insights will inform targeted strategies to mitigate frailty and promote healthy ageing globally.

## 2 Methods and materials

### 2.1 Participants

This study is a cross-sectional investigation utilizing data sourced from the NHANES, managed and implemented by the National Center for Health Statistics at the Centers for Disease Control and Prevention. NHANES employs a multi-stage, probability sampling method to select participants from a representative sample across different age, gender, racial/ethnic, and geographic categories throughout the United States. Participants provide detailed information on health status, lifestyle, and other factors through face-to-face interviews, physical examinations, and laboratory tests. Detailed information about NHANES can be obtained from its official website. This study included participants from the years 2005 to 2018. Initially, there were 30,915 participants, with exclusions made for individuals under 60 years of age (*n* = 20,340), missing lifestyle data for more than one item (*n* = 391), frailty data missing for more than 20 % (*n* = 119), resulting in a final inclusion of 10,065 participants (Supplementary Figure. S1).

### 2.2 Measures

#### 2.2.1 Life's essential 8

LE 8 is an assessment tool developed by the AHA to promote cardiovascular health and reduce the risk of cardiovascular diseases. It comprises eight indicators: diet quality, PA, nicotine exposure, sleep health, BMI, Non-HDL-C, blood glucose, and blood pressure. Each indicator is converted to a score ranging from 0 to 100 based on specific percentile points. Detailed conversion criteria have been previously described in the literature [10]. Particularly, for nicotine exposure, a score reduction of 20 is applied if exposed to secondhand smoke; for Non-HDL-C and blood pressure, a score reduction of 20 is applied if taking lipid-lowering or antihypertensive medications.

#### 2.2.2 Frailty

We used the frailty index because it captures a broader range of health deficits and is more sensitive to varying frailty levels than the frailty phenotype [13]. Frailty consists of 49 indicators [14], primarily including cognitive ability (1 item), activities of daily living (15 items), depressive symptoms (7 items), chronic diseases (13 items), healthcare service utilization (5 items), physical function and anthropometric measures (2 items), as well as laboratory tests (6 items). Detailed measurement and assessment criteria for each indicator have been reported in previous studies [14]. The frailty index is calculated by dividing the number of diseases each participant has by 49, resulting in a frailty index ranging from 0 to 1. Participants with a frailty index greater than 0.21 are defined as frail [15].

#### 2.2.3 Covariates

We included potential confounding variables. Due to the inclusion of numerous variables in LE8 and frailty (57 in total), to avoid multicollinearity and overfitting, we only included gender (male, female), age (continuous variable), education level (<high school, high school, > high school), race (Mexican American, Non-Hispanic Black, Non-Hispanic White, Other Hispanic, Other Race - Including Multi-Racial), marital status (married, single), and poverty level (continuous variable) as covariates.

### 2.3 Statistical analysis

For normally distributed continuous variables, we used mean and standard error to represent them and compared baseline differences using analysis of variance. For non-normally distributed variables, we described them using mean and quartiles and compared baseline differences using the rank-sum test. For categorical variables, case numbers (weighted percentages) were used to describe them, and baseline characteristics were compared using the Rao-Scott chi-square test. Considering the representativeness and robustness of the results, all statistical analyses were weighted. Although each indicator in LE8 was transformed into a continuous variable, considering the presence of numerous discontinuities, transforming these variables into categorical variables was deemed appropriate. Firstly, we explored the association between each component of LE8 and frailty using weighted logistic regression models and examined whether a linear relationship existed using trend tests. Secondly, to determine the dose-response relationship between each LE8 variable and frailty, we utilized logistic-based restricted cubic spline (RCS) regression to plot dose-response curves and evaluated non-linearity using likelihood ratio tests [16]. Finally, to assess the joint effect of LE8 on frailty and the priority of each LE8 component, we employed Quantile-g-computation to investigate their association with frailty [11]. In addition to the primary analyses, we conducted subgroup and sensitivity analyses. Subgroup analyses were stratified by sex (male, female) and age group (<70 years and ≥70 years) to examine whether associations between LE8 components and frailty risk varied across demographic groups. For sensitivity analyses, we adopted alternative frailty definitions using different cut-off points of the Frailty Index (FI ≥ 0.18, FI ≥ 0.25, and FI ≥ 0.25) to assess the robustness of the findings. All statistical analyses were performed using R software. A two-tailed p-value < 0.05 was considered statistically significant.

## 3 Results

### 3.1 Baseline characteristics

**Fig. 1:**
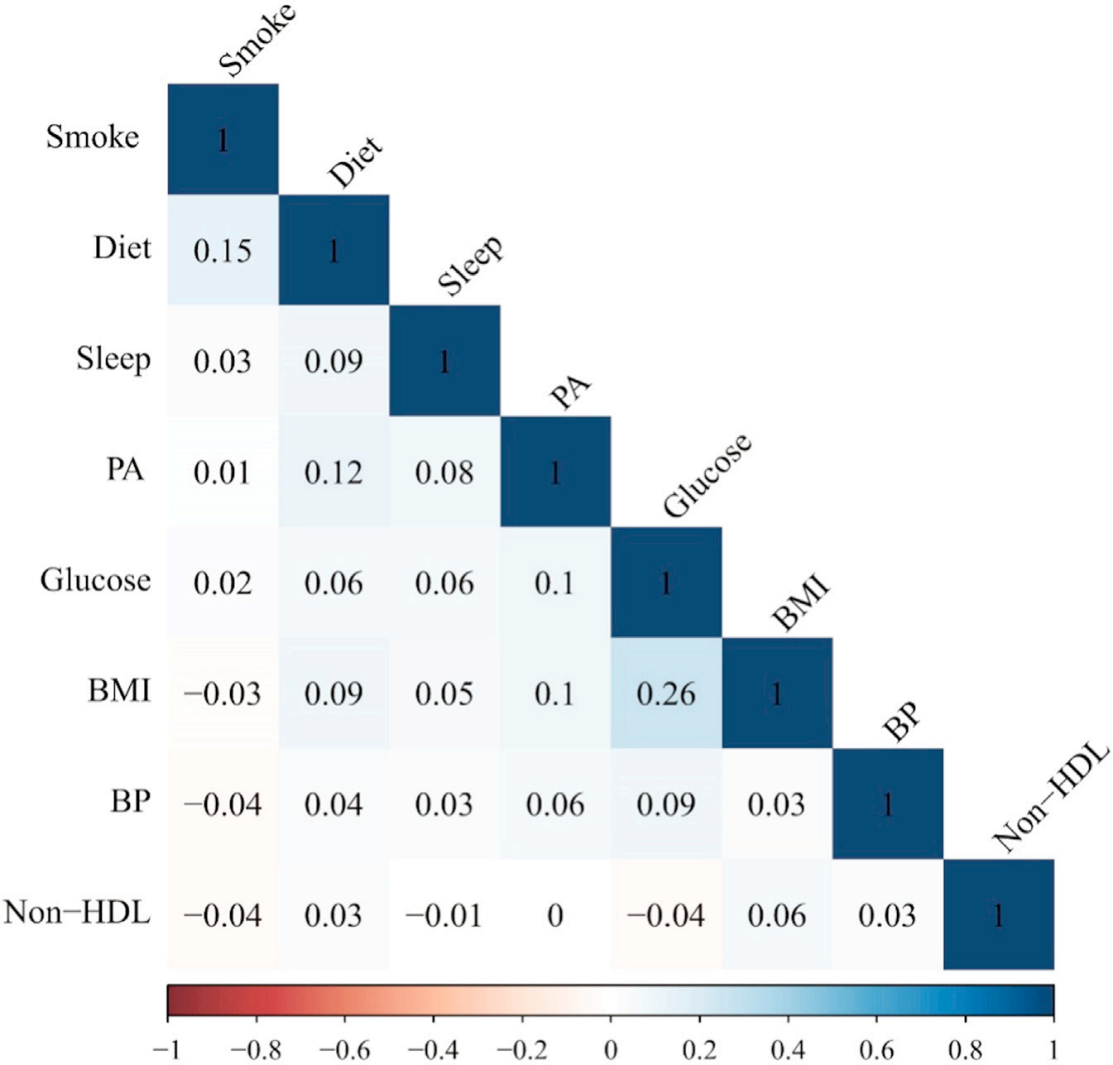
Matrix of correlation coefficients for each component of LE8.

A total of 10,065 individuals were included in the study, with a mean age of 69.61 years and a weighted proportion of 54.97 % females. Frail participants had a higher average age and lower LE8 scores compared to non-frail participants. Statistically significant differences were observed in all lifestyle components among participants grouped by frailty (*P* < .001). Frail participants were more likely to be older, single, and have lower economic status (Table 1). The results of pairwise correlations among the LE8 components indicated relatively low correlations between each indicator (Fig. 1).

**Table 1:** Survey-Weighted Characteristics of the NHANES Sample According to Frailty.

Variable	Total	Non-Frailty	Frailty	*P* value
**Age**	69.61(0.11)	68.81(0.12)	71.63(0.19)	**< 0.0001**
**Diet**	50.00(25.00,80.00)	50.00(25.00,80.00)	50.00(25.00,50.00)	**< 0.0001**
**PA**	90.00(0.00,100.00)	100.00(20.00,100.00)	0.00(0.00,100.00)	**< 0.0001**
**Smoke**	75.00(75.00,100.00)	80.00(75.00,100.00)	75.00(75.00,100.00)	**< 0.0001**
**Sleep**	100.00(70.00,100.00)	100.00(70.00,100.00)	90.00(70.00,100.00)	**< 0.0001**
**BMI**	70.00(30.00,70.00)	70.00(30.00,100.00)	30.00(15.00, 70.00)	**< 0.0001**
**HDL**	60.00(40.00,80.00)	60.00(40.00,80.00)	80.00(40.00,80.00)	**< 0.0001**
**Glucose**	60.00(60.00,100.00)	100.00(60.00,100.00)	60.00(40.00,100.00)	**< 0.0001**
**Blood pressure**	50.00(30.00,80.00)	50.00(30.00,80.00)	50.00(5.00,80.00)	**< 0.0001**
**LE 8**	64.38(54.38,73.75)	67.50(58.13,76.25)	56.25(47.50,65.00)	**< 0.0001**
**Poverty level**	3.05(1.71,5.00)	3.48(2.01,5.00)	2.08(1.25,3.65)	**< 0.0001**
**Sex**				**< 0.0001**
Female	5070(54.97)	3278(53.26)	1792(59.32)	
Male	4955(45.03)	3437(46.74)	1518(40.68)	
**Race**				**< 0.0001**
Mexican American	1181(3.65)	767(3.23)	414(4.72)	
Non-Hispanic Black	2050(7.91)	1309(6.91)	741(10.46)	
Non-Hispanic White	5284(80.96)	3584(82.77)	1700(76.34)	
Other Hispanic	895(3.13)	600(2.94)	295(3.63)	
Other Race - Including Multi-Racial	615(4.35)	455(4.16)	160(4.85)	
**Education**				**< 0.0001**
< High school	1400(6.86)	778(5.95)	622(13.80)	
High school	4765(57.13)	3512(72.17)	1253(54.69)	
> High school	2511(20.78)	1591(21.88)	920(31.51)	
**Marrital status**				**< 0.0001**
Married	5910(64.81)	4251(68.87)	1659(54.55)	
Single	4111(35.16)	2461(31.13)	1650(45.45)	

Abbreviations: LE8: Life's Essential 8; PA: physical activity; BMI: body mass index; HDL: Non-High-Density Lipoprotein Cholesterol.

### 3.2 Association between LE8 components and frailty

The association between each LE8 component and frailty risk is presented in Table 2. All components were referenced using the worst scenario (score of 0). For dietary score, participants with scores of 80 (OR=0.72, 95 % CI: 0.60–0.87) and 100 (OR=0.69, 95 % CI: 0.52–0.90) were less likely to experience frailty. Regarding physical activity, engaging in exercise was associated with a lower risk of frailty (all *P* < .05). Non-smokers were less likely to experience frailty. For sleep, participants with scores of 70 and above had a lower risk of frailty, with those scoring 100 having the lowest risk (OR=0.29, 95 % CI: 0.17–0.49). Higher BMI scores were associated with a higher likelihood of avoiding frailty. For Non-HDL-C, participants with scores of 80 (OR=1.74, 95 % CI: 1.30–2.34) and 100 (OR=1.51, 95 % CI: 1.11–2.07) were more likely to suffer from frailty. Regarding blood glucose, participants with scores of 60 (OR=0.32, 95 % CI: 0.20–0.52) and 100 (OR=0.27, 95 % CI: 0.17–0.44) were less likely to experience frailty. For blood pressure, participants with scores of 25 (OR=0.43, 95 % CI: 0.30–0.61), 50 (OR=0.51, 95 % CI: 0.37–0.69), 75 (OR=0.50, 95 % CI: 0.35–0.71), and 100 (OR=0.59, 95 % CI: 0.43–0.81) were less likely to experience frailty, while those with scores of 5, 30, and 80 were more likely to experience frailty. All LE8 components possibly exhibited linear trends (All *P* < .05).

**Table 2:** Association Between the LE8 and Risk of Frailty.

Variable	level	OR	*P* for trend
**Diet**	0		*P* = 0.005
	25	0.94 (0.79–1.13, *p*=.519)	
	50	0.96 (0.81–1.14, *p*=.633)	
	80	0.72 (0.60–0.87, *p*<.001)	
	100	0.69 (0.52–0.90, *p*=.007)	
**PA**	0		*P* < 0.001
	20	0.65 (0.45–0.93, *p*=.019)	
	40	0.62 (0.46–0.84, *p*=.002)	
	60	0.61 (0.46–0.81, *p*<.001)	
	80	0.55 (0.40–0.76, *p*<.001)	
	90	0.65 (0.49–0.87, *p*=.004)	
	100	0.40 (0.35–0.45, *p*<.001)	
**Smoke**	0		*P* < 0.001
	5	0.74 (0.24–2.28, *p*=.603)	
	25	1.15 (0.76–1.74, *p*=.501)	
	30	1.20 (0.47–3.06, *p*=.696)	
	50	1.36 (0.90–2.04, *p*=.140)	
	55	1.02 (0.65–1.60, *p*=.945)	
	75	0.85 (0.70–1.04, *p*=.123)	
	80	0.68 (0.43–1.09, *p*=.107)	
	100	0.62 (0.51–0.75, *p*<.001)	
**Sleep**	0		*P* < 0.001
	20	1.00 (0.55–1.83, *p*=.990)	
	40	0.60 (0.35–1.02, *p*=.061)	
	70	0.36 (0.21–0.61, *p*<.001)	
	90	0.43 (0.25–0.74, *p*=.002)	
	100	0.29 (0.17–0.49, *p*<.001)	
**BMI**	0		*P* < 0.001
	15	0.54 (0.40–0.73, *p*<.001)	
	30	0.42 (0.32–0.54, *p*<.001)	
	70	0.40 (0.30–0.51, *p*<.001)	
	100	0.38 (0.29–0.51, *p*<.001)	
**Non-HDL-C**	0		*P* < 0.001
	20	1.14 (0.81–1.59, *p*=.454)	
	40	1.25 (0.93–1.69, *p*=.139)	
	60	1.14 (0.84–1.56, *p*=.397)	
	80	1.74 (1.30–2.34, *p*<.001)	
	100	1.51 (1.11–2.07, *p*=.009)	
**Glucose**	0		*P* < 0.001
	10	0.77 (0.41–1.47, *p*=.433)	
	20	1.14 (0.63–2.04, *p*=.669)	
	30	0.92 (0.55–1.54, *p*=.740)	
	40	0.93 (0.57–1.52, *p*=.764)	
	60	0.32 (0.20–0.52, *p*<.001)	
	100	0.27 (0.17–0.44, *p*<.001)	
**Blood pressure**	0		*P* = 0.002
	5	1.42 (1.10–1.84, *p*=.008)	
	25	0.43 (0.30–0.61, *p*<.001)	
	30	1.31 (1.02–1.69, *p*=.034)	
	50	0.51 (0.37–0.69, *p*<.001)	
	55	1.16 (0.89–1.52, *p*=.277)	
	75	0.50 (0.35–0.71, *p*<.001)	
	80	1.45 (1.12–1.88, *p*=.005)	
	100	0.59 (0.43–0.81, *p*=.001)	

Abbreviations: PA: physical activity; BMI: body mass index; HDL: Non-High-Density Lipoprotein Cholesterol.

### 3.3 Estimation of dose-response relationship between LE8 and frailty

**Fig. 2:**
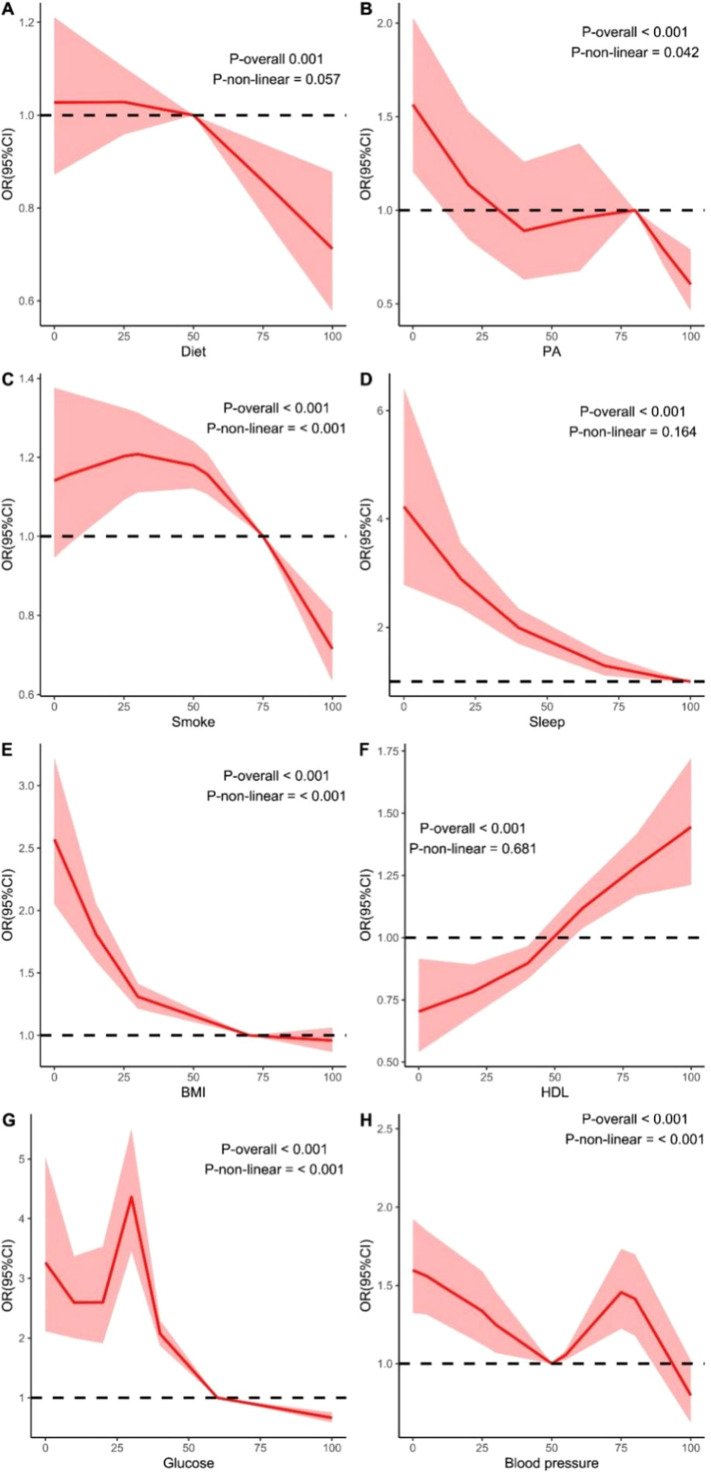
logistic-based restricted cubic spline regression for each component of LE8.

To estimate dose-response curves and assess non-linear trends, we conducted RCS analysis. As shown in Fig. 2, the results revealed that there was no linear association between diet, exercise, Non-HDL-C, and sleep with frailty risk (*P* > .05). Smoking, BMI, blood glucose, and blood pressure possibly exhibited non-linear relationships with frailty risk (All *P* < .05).

### 3.4 Joint effects of LE8 on frailty and importance of each component

**Fig. 3:**
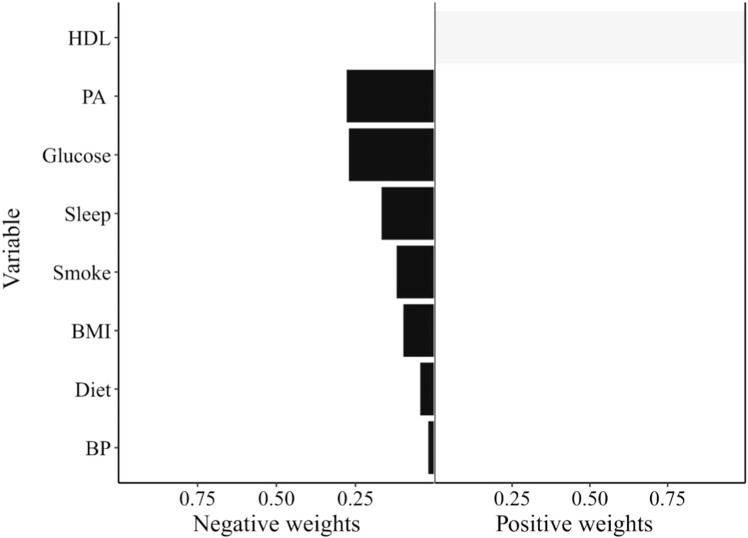
Prioritization of LE8 components based on Quantile-g-computation.

After adjusting for confounding factors, the risk of frailty was reduced by 94 % (95 % CI: 92 %−95 %) when the 8 lifestyle factors were increased by one level at the same time. In Fig. 3, only Non-HDL-C had a positive weight, while the other 7 lifestyle factors had negative weights. Among the negative weights, PA had the highest weight, followed by blood glucose, sleep, smoking, BMI, diet, and blood pressure.

### 3.5 Subgroup analysis and sensitivity analyses

Subgroup analysis showed consistent directions of associations across sex and age groups. For example, higher diet scores were associated with lower frailty risk in both males and females (e.g., diet score of 80: OR = 0.77, 95 % CI: 0.61–0.98 in males; OR = 0.75, 95 % CI: 0.58–0.95 in females). Among individuals aged <70 years, protective effects of certain lifestyle behaviors, such as healthy diet and optimal blood glucose levels, appeared more pronounced (Supplementary Table S1). Sensitivity analyses using alternative frailty definitions yielded similar results. Regardless of the cut-off used (FI ≥ 0.18, FI ≥ 0.25, or FI ≥ 0.25 with five common chronic diseases excluded), the direction and significance of associations between LE8 components and frailty remained stable, indicating the robustness of our findings (Supplementary Table S2).

## 4 Discussion

This study evaluated the association between LE8 and frailty using data from seven cycles of NHANES. Specifically, it examined: 1) the association and dose-response relationship between individual lifestyle factors and frailty, and 2) the joint effect of LE8 on frailty and the importance of each indicator. The results indicated that lower levels of Non-HDL-C may be a risk factor, while higher scores in other indicators of LE8 were associated with a reduced risk of frailty. Smoking, BMI, blood glucose, and blood pressure showed potential non-linear relationships with frailty risk. Among factors negatively associated with frailty, PA, blood glucose, and sleep were the most important.

Previous studies have examined the association between some lifestyle factors and frailty, but these have often been limited in scope. Few have investigated the association between frailty and lifestyle based on LE8. For instance, a Finnish cohort study examined the association between six lifestyle factors (exercise, diet, sleep, alcohol, smoking, and body composition) and frailty, finding that a greater number of lifestyle factors was associated with lower frailty levels, consistent with our findings [17]. Although previous studies explored the association between Life’s Sample 7 and frailty, they focused on the overall score, which may lack specificity for individual and public health practices [18]. Our study addressed these gaps, providing observational evidence that may inform both individuals and policymakers on potential strategies to mitigate frailty risk.

Among the lifestyle factors negatively associated with frailty, PA, blood glucose, and sleep emerged as the most important. PA is a recognized protective factor, potentially improving mental health and reducing depression, which is part of frailty [19]. Research indicates that exercise can alleviate depression through neurobiological mechanisms, including upregulating the expression of neurotrophic factors such as BDNF and reducing inflammatory signaling pathways [20]. These pathways may help explain the observed associations between PA and lower frailty risk. Additionally, PA is associated with healthier BMI levels, which in turn relate to lower frailty prevalence. However, some randomized controlled trials have reported potential adverse events in older adults, such as muscle pain and fatigue, which may attenuate the net benefits of exercise [21,22]. Therefore, further research is warranted to identify appropriate types and intensities of physical activity that are most beneficial for frailty prevention. Similarly, elevated blood glucose levels may be associated with increased frailty risk [23,24]. Higher blood glucose levels or diabetes may impact frailty through several potential pathways, including increased risk of chronic inflammation, chronic hyperglycemia-induced microvascular damage, and skeletal muscle mitochondrial dysfunction [[25], [26], [27]]. The observed non-linear association between blood glucose and frailty may suggest a U-shaped relationship, consistent with prior findings [24]. Sleep health is also a crucial factor in frailty. Poor sleep quality or shorter sleep duration may lead to reduced basal metabolic rate and energy expenditure, compromising muscle mass and immune function, thereby increasing susceptibility to diseases and functional decline [21,28,29]. Furthermore, disrupted circadian rhythms due to sleep disturbances may exacerbate frailty through oxidative and neuronal damage [29]. These associations highlight the importance of maintaining healthy sleep patterns, but additional longitudinal studies are needed to explore these links further.

A surprising finding in this study was the association between lower Non-HDL-C levels and higher frailty risk. Currently, there is limited research on the detailed association between Non-HDL-C and frailty, especially lacking longitudinal data. On one hand, low Non-HDL-C levels imply high HDL-C levels, which may indicate lower cardiovascular or mortality risks [30]. Thus, the association between low Non-HDL-C and frailty may be due to fewer frail individuals in the higher Non-HDL-C group, resulting in reverse causation. Therefore, caution is needed when interpreting the association between Non-HDL-C and frailty, requiring higher-level evidence from cohort studies or RCTs to guide public health practice.

Another intriguing finding is the association between higher frailty risk and individuals taking antihypertensive medication to maintain relatively normal or healthy blood pressure levels [31]. This phenomenon suggests that frailty caused by hypertension may be irreversible. One potential benefit of antihypertensive medication is reducing cardiovascular disease or mortality. However, some previous studies have suggested that antihypertensive drugs may increase the risk of other complications in older adults, such as falls, cognitive impairment, orthostatic hypotension, and pressure ulcers [[32], [33], [34], [35]]. Further investigations are needed to assess optimal treatment strategies for this group and to guide clinical decision-making.

This study has several strengths. It is among the first to apply the Quantile-g-computation method to evaluate the joint effects and relative importance of LE8 components in relation to frailty. We used data from a large, nationally representative sample, and applied rigorous statistical models with multiple sensitivity and subgroup analyses to ensure robustness. Nonetheless, several limitations should be noted. Firstly, this study is cross-sectional, unable to determine causality. Therefore, more rigorous designs and measurements are needed to replicate the research and ascertain the association between LE8 and frailty. Secondly, due to some self-reported variables in LE8, measurement errors and potential unmeasured confounding are unavoidable. Nevertheless, we conducted rigorous statistical analyses to control confounding and ensure reasonable estimation results. Lastly, as the study selected adults aged 60 and above, the sample may not represent all age groups in the United States. Additionally, the study results may not necessarily be applicable to other racial groups outside the United States.

## 5 Conclusion

The study findings suggest that various components of LE8 are associated with frailty, with physical exercise, blood glucose, and sleep emerging as potential key lifestyle factors. Future evidence-based prospective studies are needed to assess the association between LE8 and frailty and determine the priority of each LE8 component to provide personalized recommendations for delaying frailty.

## ETHICS approval and consent to participate

The U.S. National Centre for Health Statistics Ethics Review Board approved the NHANES survey protocols (Protocol #2005-06 and Protocol #2011-2017), and the study complied with the Declaration of Helsinki. All participants gave written informed consent.

## Consent for publication

Not applicable.

## Availability of data and materials

This study used public data at https://www.cdc.gov/nchs/nhanes/index.htm

## Funding

There was not the funding.

## CRediT authorship contribution statement

**Yuanyuan Wu:** Writing – original draft, Visualization, Data curation. **Hongyan Peng:** Writing – original draft, Visualization, Data curation. **Rui Xu:** Visualization, Validation. **Yingxue Hua:** Writing – review & editing. **Yanan Zhang:** Writing – review & editing, Supervision.

## Conflict of interest

we confirm that all authors have no conflicts of interest to declare related to the submitted manuscript. Neither the research reported in this article nor any of the authors have received any financial or non-financial support, benefits, or other potential interests from commercial parties directly or indirectly associated with the manuscript’s subject matter.

## Appendix Supplementary materials

Image, application 1
