# Supplementary material for: Association of lifestyle modifications with frailty in older adults: A cross-sectional study using NHANES
Source: J Frailty Aging. 2025 Jun 30;14(4):100061. doi: 10.1016/j.tjfa.2025.100061 (PMC12399231; doi:10.1016/j.tjfa.2025.100061)
Supplement: Supplementary file 1 [file mmc1.docx]

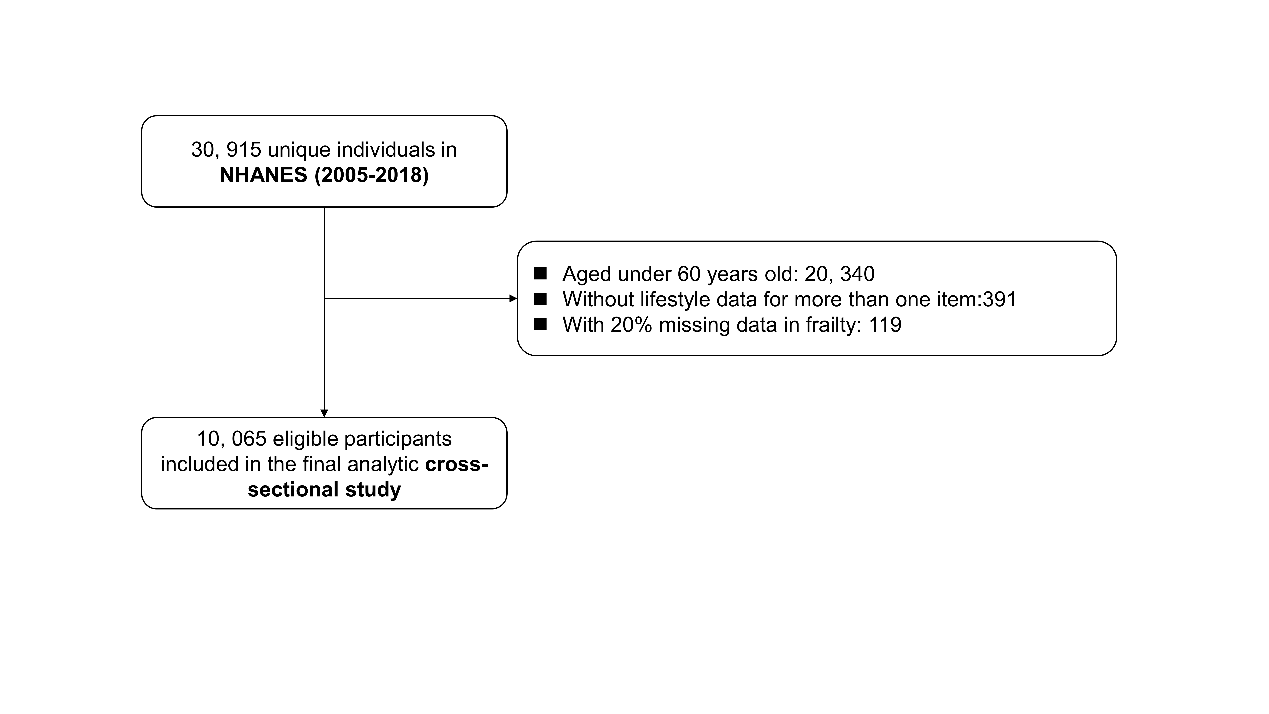


**Figure S1. Flow chart of sample selection**

**Table S1. Association Between the LE8 and Risk of Frailty group by sex and age.**

|  |  | **Female** | **Male** | **Yonger (Aged <70)** | **Older (Aged ≥70)** |
| --- | --- | --- | --- | --- | --- |
| **Variable** | **level** | **OR (95% CI)** | **OR (95% CI)** | **OR (95% CI)** | **OR (95% CI)** |
| **Diet** | 0 |  |  |  |  |
|  | 25 | 1.11 (0.88-1.41, p=.380) | 0.87 (0.69-1.09, p=.219) | 0.93 (0.74-1.18, p=.558) | 1.00 (0.79-1.26, p=.974) |
|  | 50 | 0.97 (0.77-1.22, p=.814) | 1.01 (0.80-1.26, p=.955) | 0.88 (0.70-1.11, p=.280) | 1.09 (0.87-1.37, p=.440) |
|  | 80 | 0.77 (0.61-0.98, p=.033) | 0.75 (0.58-0.95, p=.018) | 0.65 (0.50-0.84, p<.001) | 0.85 (0.68-1.08, p=.180) |
|  | 100 | 0.68 (0.48-0.97, p=.031) | 0.82 (0.55-1.22, p=.330) | 0.51 (0.33-0.78, p=.002) | 0.92 (0.66-1.27, p=.601) |
| **PA** | 0 |  |  |  |  |
|  | 20 | 0.85 (0.53-1.35, p=.486) | 0.64 (0.38-1.10, p=.106) | 0.74 (0.44-1.25, p=.263) | 0.79 (0.50-1.26, p=.324) |
|  | 40 | 0.61 (0.41-0.91, p=.016) | 0.60 (0.40-0.89, p=.011) | 0.71 (0.47-1.09, p=.117) | 0.52 (0.36-0.76, p<.001) |
|  | 60 | 0.59 (0.41-0.84, p=.004) | 0.66 (0.44-0.99, p=.044) | 0.66 (0.44-0.99, p=.045) | 0.54 (0.38-0.78, p=.001) |
|  | 80 | 0.51 (0.33-0.77, p=.002) | 0.59 (0.39-0.89, p=.012) | 0.56 (0.36-0.87, p=.009) | 0.47 (0.31-0.71, p<.001) |
|  | 90 | 0.74 (0.52-1.06, p=.100) | 0.65 (0.44-0.96, p=.030) | 0.75 (0.51-1.10, p=.140) | 0.65 (0.45-0.94, p=.021) |
|  | 100 | 0.44 (0.37-0.53, p<.001) | 0.38 (0.32-0.46, p<.001) | 0.46 (0.39-0.56, p<.001) | 0.36 (0.30-0.42, p<.001) |
| **Smoke** | 0 |  |  |  |  |
|  | 5 | 1.01 (0.20-5.06, p=.987) | 0.67 (0.18-2.49, p=.545) | 3.51 (0.83-14.76, p=.087) | 0.24 (0.04-1.36, p=.106) |
|  | 25 | 1.22 (0.65-2.26, p=.539) | 0.80 (0.50-1.29, p=.363) | 1.05 (0.54-2.03, p=.889) | 0.93 (0.58-1.50, p=.771) |
|  | 30 | 1.81 (0.45-7.29, p=.401) | 0.91 (0.31-2.65, p=.857) | 0.98 (0.38-2.55, p=.967) | 1.64 (0.22-12.19, p=.628) |
|  | 50 | 1.19 (0.65-2.17, p=.566) | 1.68 (1.02-2.75, p=.040) | 1.94 (1.22-3.09, p=.005) | 0.81 (0.41-1.62, p=.557) |
|  | 55 | 0.71 (0.33-1.51, p=.376) | 1.11 (0.66-1.87, p=.685) | 1.05 (0.59-1.89, p=.857) | 0.88 (0.47-1.66, p=.692) |
|  | 75 | 0.81 (0.61-1.08, p=.154) | 0.84 (0.65-1.07, p=.157) | 1.04 (0.81-1.33, p=.751) | 0.75 (0.56-1.00, p=.052) |
|  | 80 | 0.76 (0.44-1.31, p=.329) | 0.55 (0.27-1.14, p=.107) | 0.73 (0.40-1.31, p=.290) | 0.70 (0.38-1.29, p=.249) |
|  | 100 | 0.62 (0.48-0.81, p<.001) | 0.60 (0.46-0.78, p<.001) | 0.61 (0.48-0.77, p<.001) | 0.64 (0.48-0.86, p=.003) |
| **Sleep** | 0 |  |  |  |  |
|  | 20 | 0.74 (0.34-1.64, p=.463) | 1.49 (0.68-3.27, p=.325) | 1.96 (0.96-3.97, p=.063) | 0.45 (0.17-1.14, p=.091) |
|  | 40 | 0.39 (0.19-0.81, p=.011) | 0.91 (0.46-1.82, p=.794) | 1.02 (0.54-1.91, p=.953) | 0.27 (0.12-0.65, p=.003) |
|  | 70 | 0.25 (0.12-0.52, p<.001) | 0.58 (0.29-1.16, p=.126) | 0.57 (0.31-1.07, p=.082) | 0.21 (0.09-0.49, p<.001) |
|  | 90 | 0.28 (0.13-0.58, p<.001) | 0.60 (0.29-1.21, p=.151) | 0.76 (0.39-1.46, p=.408) | 0.18 (0.08-0.43, p<.001) |
|  | 100 | 0.20 (0.10-0.41, p<.001) | 0.45 (0.23-0.88, p=.020) | 0.47 (0.26-0.87, p=.015) | 0.15 (0.06-0.35, p<.001) |
| **BMI** | 0 |  |  |  |  |
|  | 15 | 0.60 (0.43-0.85, p=.004) | 0.54 (0.34-0.86, p=.009) | 0.68 (0.48-0.97, p=.032) | 0.49 (0.31-0.78, p=.002) |
|  | 30 | 0.44 (0.32-0.60, p<.001) | 0.42 (0.28-0.63, p<.001) | 0.43 (0.32-0.59, p<.001) | 0.47 (0.31-0.72, p<.001) |
|  | 70 | 0.41 (0.30-0.56, p<.001) | 0.38 (0.25-0.57, p<.001) | 0.37 (0.27-0.51, p<.001) | 0.46 (0.31-0.69, p<.001) |
|  | 100 | 0.33 (0.24-0.46, p<.001) | 0.43 (0.28-0.66, p<.001) | 0.35 (0.25-0.49, p<.001) | 0.45 (0.30-0.69, p<.001) |
| **Non-HDL-C** | 0 |  |  |  |  |
|  | 20 | 1.01 (0.68-1.49, p=.962) | 0.96 (0.57-1.63, p=.889) | 0.96 (0.63-1.47, p=.869) | 1.04 (0.65-1.66, p=.861) |
|  | 40 | 0.97 (0.68-1.37, p=.849) | 1.39 (0.87-2.22, p=.168) | 0.93 (0.63-1.36, p=.709) | 1.37 (0.91-2.07, p=.135) |
|  | 60 | 1.01 (0.70-1.46, p=.956) | 0.95 (0.58-1.54, p=.823) | 0.94 (0.63-1.40, p=.765) | 1.06 (0.69-1.63, p=.803) |
|  | 80 | 1.36 (0.96-1.94, p=.084) | 1.75 (1.11-2.76, p=.017) | 1.45 (0.99-2.13, p=.056) | 1.71 (1.14-2.56, p=.009) |
|  | 100 | 1.26 (0.86-1.84, p=.230) | 1.40 (0.87-2.25, p=.172) | 1.18 (0.79-1.77, p=.413) | 1.53 (1.00-2.34, p=.053) |
| **Glucose** | 0 |  |  |  |  |
|  | 10 | 1.17 (0.48-2.83, p=.727) | 0.63 (0.27-1.44, p=.270) | 1.16 (0.57-2.35, p=.680) | 0.41 (0.13-1.31, p=.132) |
|  | 20 | 1.29 (0.58-2.89, p=.534) | 1.30 (0.63-2.69, p=.471) | 0.98 (0.51-1.89, p=.961) | 1.58 (0.57-4.35, p=.380) |
|  | 30 | 1.15 (0.56-2.33, p=.706) | 0.80 (0.41-1.54, p=.496) | 0.98 (0.55-1.74, p=.939) | 0.80 (0.32-2.03, p=.642) |
|  | 40 | 1.29 (0.66-2.52, p=.455) | 0.93 (0.50-1.74, p=.827) | 1.22 (0.71-2.10, p=.475) | 0.90 (0.37-2.22, p=.826) |
|  | 60 | 0.34 (0.18-0.66, p=.001) | 0.37 (0.20-0.68, p=.002) | 0.36 (0.21-0.61, p<.001) | 0.30 (0.12-0.73, p=.008) |
|  | 100 | 0.30 (0.16-0.59, p<.001) | 0.33 (0.18-0.61, p<.001) | 0.38 (0.22-0.63, p<.001) | 0.24 (0.10-0.59, p=.002) |
| **Blood pressure** | 0 |  |  |  |  |
|  | 5 | 1.42 (1.04-1.94, p=.026) | 1.51 (1.01-2.24, p=.043) | 1.81 (1.15-2.85, p=.011) | 1.35 (1.01-1.80, p=.045) |
|  | 25 | 0.46 (0.30-0.71, p<.001) | 0.40 (0.24-0.67, p<.001) | 0.54 (0.32-0.92, p=.024) | 0.37 (0.24-0.58, p<.001) |
|  | 30 | 1.17 (0.86-1.59, p=.305) | 1.29 (0.88-1.90, p=.197) | 1.43 (0.93-2.22, p=.106) | 1.15 (0.86-1.54, p=.345) |
|  | 50 | 0.48 (0.32-0.71, p<.001) | 0.51 (0.32-0.79, p=.003) | 0.68 (0.42-1.09, p=.108) | 0.35 (0.23-0.52, p<.001) |
|  | 55 | 1.10 (0.80-1.52, p=.562) | 1.30 (0.87-1.95, p=.206) | 1.66 (1.05-2.62, p=.029) | 0.93 (0.68-1.26, p=.629) |
|  | 75 | 0.43 (0.28-0.67, p<.001) | 0.64 (0.39-1.06, p=.081) | 0.73 (0.43-1.24, p=.243) | 0.38 (0.24-0.60, p<.001) |
|  | 80 | 1.26 (0.91-1.74, p=.158) | 1.78 (1.20-2.63, p=.004) | 1.75 (1.12-2.74, p=.014) | 1.38 (1.02-1.86, p=.034) |
|  | 100 | 0.48 (0.32-0.72, p<.001) | 0.72 (0.46-1.14, p=.162) | 0.73 (0.45-1.18, p=.200) | 0.52 (0.35-0.79, p=.002) |

Abbreviations: PA: physical activity; BMI: body mass index; HDL: Non-High-Density Lipoprotein Cholesterol.

**Table S2. Sensitivity Analyses for the Association Between the LE8 and Risk of Frailty.**

|  |  | **FI >= 0.18** | **FI >= 0.25** | **FI >= 0.25** |
| --- | --- | --- | --- | --- |
| **Variable** | **level** | **OR (95% CI)** | **OR (95% CI)** | **OR (95% CI)** |
| **Diet** | 0 |  |  |  |
|  | 25 | 0.87 (0.73-1.04, p=.124) | 0.94 (0.77-1.14, p=.523) | 0.97 (0.77-1.22, p=.811) |
|  | 50 | 0.91 (0.77-1.08, p=.275) | 0.92 (0.76-1.12, p=.415) | 1.04 (0.84-1.30, p=.709) |
|  | 80 | 0.71 (0.59-0.84, p<.001) | 0.73 (0.59-0.89, p=.002) | 0.83 (0.65-1.06, p=.131) |
|  | 100 | 0.57 (0.44-0.73, p<.001) | 0.73 (0.54-1.00, p=.051) | 0.77 (0.52-1.13, p=.181) |
| **PA** | 0 |  |  |  |
|  | 20 | 0.75 (0.53-1.06, p=.101) | 0.67 (0.45-1.00, p=.049) | 0.62 (0.39-1.00, p=.051) |
|  | 40 | 0.56 (0.42-0.75, p<.001) | 0.44 (0.31-0.63, p<.001) | 0.35 (0.22-0.56, p<.001) |
|  | 60 | 0.57 (0.43-0.74, p<.001) | 0.53 (0.38-0.73, p<.001) | 0.49 (0.33-0.73, p<.001) |
|  | 80 | 0.50 (0.37-0.67, p<.001) | 0.51 (0.36-0.74, p<.001) | 0.46 (0.29-0.72, p<.001) |
|  | 90 | 0.60 (0.46-0.79, p<.001) | 0.66 (0.48-0.92, p=.012) | 0.67 (0.46-0.98, p=.039) |
|  | 100 | 0.44 (0.39-0.50, p<.001) | 0.39 (0.34-0.46, p<.001) | 0.35 (0.29-0.42, p<.001) |
| **Smoke** | 0 |  |  |  |
|  | 5 | 0.57 (0.19-1.71, p=.315) | 1.36 (0.44-4.20, p=.594) | 1.25 (0.37-4.29, p=.717) |
|  | 25 | 0.90 (0.60-1.36, p=.621) | 1.10 (0.71-1.70, p=.668) | 0.76 (0.46-1.28, p=.303) |
|  | 30 | 1.23 (0.49-3.09, p=.653) | 1.28 (0.47-3.48, p=.635) | 0.97 (0.29-3.22, p=.965) |
|  | 50 | 1.02 (0.68-1.52, p=.928) | 1.18 (0.76-1.83, p=.467) | 1.08 (0.65-1.78, p=.764) |
|  | 55 | 0.79 (0.51-1.24, p=.314) | 1.10 (0.68-1.79, p=.691) | 0.82 (0.46-1.45, p=.495) |
|  | 75 | 0.86 (0.71-1.05, p=.134) | 0.83 (0.67-1.04, p=.104) | 0.73 (0.56-0.93, p=.013) |
|  | 80 | 0.58 (0.37-0.90, p=.015) | 0.63 (0.38-1.07, p=.086) | 0.44 (0.23-0.85, p=.015) |
|  | 100 | 0.61 (0.51-0.74, p<.001) | 0.61 (0.49-0.75, p<.001) | 0.52 (0.40-0.67, p<.001) |
| **Sleep** | 0 |  |  |  |
|  | 20 | 1.03 (0.55-1.94, p=.918) | 0.83 (0.46-1.50, p=.544) | 0.84 (0.45-1.56, p=.572) |
|  | 40 | 0.52 (0.30-0.92, p=.024) | 0.54 (0.32-0.93, p=.026) | 0.52 (0.30-0.92, p=.024) |
|  | 70 | 0.37 (0.21-0.64, p<.001) | 0.32 (0.19-0.55, p<.001) | 0.36 (0.20-0.62, p<.001) |
|  | 90 | 0.43 (0.24-0.77, p=.004) | 0.38 (0.22-0.66, p<.001) | 0.39 (0.22-0.69, p=.002) |
|  | 100 | 0.29 (0.17-0.51, p<.001) | 0.26 (0.15-0.44, p<.001) | 0.26 (0.15-0.45, p<.001) |
| **BMI** | 0 |  |  |  |
|  | 15 | 0.56 (0.41-0.76, p<.001) | 0.60 (0.44-0.82, p=.001) | 0.69 (0.49-0.97, p=.033) |
|  | 30 | 0.47 (0.36-0.62, p<.001) | 0.46 (0.35-0.61, p<.001) | 0.55 (0.41-0.74, p<.001) |
|  | 70 | 0.46 (0.35-0.60, p<.001) | 0.42 (0.32-0.55, p<.001) | 0.45 (0.33-0.60, p<.001) |
|  | 100 | 0.40 (0.30-0.53, p<.001) | 0.42 (0.32-0.56, p<.001) | 0.46 (0.34-0.64, p<.001) |
| **Non-HDL-C** | 0 |  |  |  |
|  | 20 | 1.01 (0.74-1.37, p=.971) | 1.05 (0.72-1.51, p=.812) | 0.93 (0.61-1.42, p=.734) |
|  | 40 | 1.21 (0.91-1.59, p=.188) | 0.99 (0.71-1.38, p=.944) | 0.80 (0.55-1.18, p=.267) |
|  | 60 | 1.08 (0.81-1.44, p=.618) | 0.96 (0.68-1.36, p=.808) | 0.69 (0.46-1.04, p=.078) |
|  | 80 | 1.72 (1.31-2.28, p<.001) | 1.45 (1.05-2.01, p=.023) | 1.18 (0.81-1.71, p=.384) |
|  | 100 | 1.34 (1.00-1.80, p=.048) | 1.23 (0.87-1.74, p=.237) | 1.08 (0.72-1.61, p=.713) |
| **Glucose** | 0 |  |  |  |
|  | 10 | 0.76 (0.39-1.47, p=.408) | 0.60 (0.31-1.15, p=.126) | 0.47 (0.23-0.95, p=.035) |
|  | 20 | 1.22 (0.66-2.26, p=.522) | 0.76 (0.42-1.36, p=.354) | 0.65 (0.35-1.21, p=.175) |
|  | 30 | 0.96 (0.56-1.66, p=.896) | 0.79 (0.47-1.33, p=.374) | 0.64 (0.37-1.10, p=.108) |
|  | 40 | 0.96 (0.57-1.61, p=.872) | 0.87 (0.53-1.43, p=.583) | 0.72 (0.43-1.20, p=.202) |
|  | 60 | 0.31 (0.19-0.51, p<.001) | 0.28 (0.17-0.46, p<.001) | 0.23 (0.14-0.39, p<.001) |
|  | 100 | 0.26 (0.16-0.43, p<.001) | 0.27 (0.17-0.44, p<.001) | 0.21 (0.13-0.35, p<.001) |
| **Blood pressure** | 0 |  |  |  |
|  | 5 | 1.34 (1.04-1.73, p=.024) | 1.25 (0.94-1.66, p=.118) | 1.19 (0.85-1.65, p=.306) |
|  | 25 | 0.46 (0.34-0.64, p<.001) | 0.44 (0.29-0.65, p<.001) | 0.55 (0.34-0.89, p=.015) |
|  | 30 | 1.26 (0.98-1.61, p=.070) | 1.28 (0.97-1.69, p=.077) | 1.27 (0.92-1.76, p=.141) |
|  | 50 | 0.44 (0.33-0.58, p<.001) | 0.44 (0.31-0.63, p<.001) | 0.37 (0.23-0.61, p<.001) |
|  | 55 | 1.22 (0.94-1.58, p=.142) | 1.11 (0.83-1.48, p=.496) | 1.10 (0.78-1.56, p=.568) |
|  | 75 | 0.48 (0.35-0.66, p<.001) | 0.55 (0.36-0.82, p=.003) | 0.40 (0.23-0.70, p=.001) |
|  | 80 | 1.40 (1.08-1.80, p=.010) | 1.30 (0.98-1.72, p=.070) | 1.22 (0.87-1.70, p=.244) |
|  | 100 | 0.53 (0.40-0.71, p<.001) | 0.62 (0.43-0.89, p=.009) | 0.67 (0.43-1.04, p=.075) |

Abbreviations: PA: physical activity; BMI: body mass index; HDL: Non-High-Density Lipoprotein Cholesterol.
